# Effect of Switching to the Tobacco Heating System Versus Continued Cigarette Smoking on Chronic Generalized Periodontitis Treatment Outcome: Protocol for a Randomized Controlled Multicenter Study

**DOI:** 10.2196/15350

**Published:** 2021-01-18

**Authors:** Sandrine Pouly, Wee Teck Ng, Muriel Benzimra, Alexandre Soulan, Nicolas Blanc, Filippo Zanetti, Patrick Picavet, Gizelle Baker, Christelle Haziza

**Affiliations:** 1 Philip Morris Products SA Neuchâtel Switzerland

**Keywords:** smoking, tobacco, periodontitis, oral health, Tobacco Heating System, modified risk tobacco product, clinical attachment level, probing depth, periodontal pocket

## Abstract

**Background:**

Smoking is a significant risk factor for periodontal disease and tooth loss, as shown in several clinical studies comparing smokers and nonsmokers. Although only a few longitudinal studies have assessed the outcome of periodontal disease after smoking cessation, they indicated that recovery after nonsurgical treatment was more successful in those who had quit smoking. As part of tobacco harm reduction strategies, substituting cigarettes with alternative, less harmful tobacco products is an approach complementary to cessation for smokers who would otherwise continue to smoke. The Tobacco Heating System (THS), developed by Philip Morris International (commercialized as IQOS), is part of the heat-not-burn product category. The IQOS device electrically heats tobacco instead of burning it, at much lower temperatures than cigarettes, thereby producing substantially lower levels of harmful and potentially harmful constituents, while providing the nicotine, taste, ritual, and a sensory experience that closely parallel those of cigarettes. Phillip Morris International has published the results from a broad clinical assessment program, which was established to scientifically substantiate the harm reduction potential of the THS among adult healthy smokers switching to the THS. The program is now progressing toward including adult smokers with smoking-related diseases.

**Objective:**

The goal of this study is to demonstrate favorable changes of periodontal endpoints in response to mechanical periodontal therapy in patients with generalized chronic periodontitis who completely switched to THS use compared with continued cigarette smoking.

**Methods:**

This is a randomized controlled two-arm parallel-group multicenter Japanese study conducted in patients with chronic generalized periodontitis who switch from cigarettes to THS compared with smokers continuing to smoke cigarettes for 6 months. The patients were treated with mechanical periodontal therapy as per standard of care in Japan. The primary objective of the study is to demonstrate the beneficial effect of switching to THS use compared with continued cigarette smoking on pocket depth (PD) reduction in all sites with an initial PD≥4 mm. The secondary objectives include evaluation of other periodontal parameters (eg, clinical attachment level or gingival inflammation) and overall oral health status upon switching to THS. Safety was monitored throughout the study.

**Results:**

In total, 172 subjects were randomized to the cigarette (n=86) or THS (n=86) groups, and all 172 completed the study. The conduct phase of the study is completed, while data cleaning and analyses are ongoing.

**Conclusions:**

This study is the first to test a heat-not-burn tobacco product in smokers with an already established disease. The results should further strengthen the evidence that switching to THS can significantly reduce the risk of smoking-related diseases if favorable changes in the evolution of chronic generalized periodontitis after mechanical therapy are found when compared with continued cigarette smoking.

**Trial Registration:**

ClinicalTrials.gov NCT03364751; https://clinicaltrials.gov/ct2/show/NCT03364751

**International Registered Report Identifier (IRRID):**

DERR1-10.2196/15350

## Introduction

Smokers are at higher risk of developing periodontal diseases [1-4]. This association has been shown in numerous studies, including evidence of a dose-response relationship between smoking intensity and the risk for periodontitis, as both the number of cigarettes smoked and the duration of smoking are positively associated with disease risk [4-6]. The magnitude of the relative risk estimated for periodontal disease associated with smoking varies from 1.4 to 5.0 in different studies [4]. In a Japanese study, the odds ratio of having periodontitis among current smokers compared with those who had never smoked was 1.74 [7].

The pathophysiological mechanisms involved in the increase of periodontal disease prevalence in active smokers mainly involve alteration of the inflammatory host response. Specifically, an increased release of inflammatory mediators occurs due to chronic exposure to the harmful and potentially harmful constituents (HPHCs) of cigarette smoke produced by the combustion of tobacco [8,9]. The inflammation not only contributes to tissue damage but also negatively impacts the reparative and regenerative potential of the periodontium and the cell lining of the oral cavity in general [1,10]. The presence of proinflammatory cytokines can be assessed from the gingival crevicular fluid collected from the pockets of diseased teeth, as described by Tymkiw et al [10], and serves as a quantifiable marker of inflammation. Additionally, smoking has been shown to cause dose-dependent quantitative and qualitative changes in the subgingival microflora (ie, increased abundance of *Porphyromonas gingivalis* and *Tannerella forsythia*), which also contribute to creating an unfavorable environment for the periodontium [11]. However, the exact understanding of periodontal microbiology is still evolving [12].

In chronic periodontitis, clinical parameters, including periodontal pocket depth (PD) and clinical attachment level (CAL), were found to be increased in smokers compared with those of nonsmokers (reviewed in [13]). Conversely, bleeding on probing (BOP), erythema, edema, and the inflammatory response associated with plaque accumulation have been shown to be less pronounced or delayed in smokers compared with those of never smokers [14-18]. Several studies have assessed the differences of periodontal parameters between smokers and former smokers, which clearly suggest that smoking cessation is beneficial for subjects undergoing therapy for chronic periodontitis [13,15,19-22]. Only two prospective studies could be identified that followed smokers after smoking cessation [23,24]. Although the results showed some benefits of quitting, the study particularly highlighted the difficulty of conducting studies of this nature owing to high dropout rates and low compliance in completing cessation. Alternatively, asking subjects in a dental setting to switch to nicotine-containing products that are likely to be safer alternatives than smoking cigarettes seems to be an avenue that may be more successful, as suggested by the pilot study of Holliday et al [25]. In all studies, the most consistent findings were favorable changes in PD in nonsmokers or former smokers, whereas changes in CAL were hardly ever found to be statistically significant.

The role of nicotine in the development and maintenance of periodontitis is not clear [26,27]. Available literature suggests that nicotine affects gingival blood flow, cytokine production, and neutrophil and other immune cell function [28]. Although nicotine replacement therapy (NRT) is part of smoking cessation programs, it has not been reported as a major issue for periodontitis to date. Similarly, the first studies on e-cigarettes and oral health could not conclusively demonstrate a negative role of nicotine on the improvement of periodontitis [29,30], although both smoking cessation [17] and switching to e-cigarettes [31] have been associated with increased BOP. Thus, an increase in BOP might simply reflect the effect of decreasing smoke toxicants rather than an effect of vaping itself.

The first line of treatment in periodontal diseases is to restore a healthy periodontium by mechanically removing supra- and subgingival plaque and calculus deposits (an intervention called scaling and root planing [SRP]), with or without antibiotic treatment [32]. In more severe cases, or cases that do not resolve after nonsurgical intervention, surgery is usually performed. Patients are also instructed on how to improve their oral hygiene. Per the recommendations of dental and health care associations such as the World Health Organization [33,34] and Japanese Society of Periodontology [35], patients should be informed of the importance of primary prevention of tobacco use and on smoking cessation programs, which would also contribute as an intervention for oral health diseases [36,37].

Philip Morris International (PMI) develops, assesses, and commercializes a portfolio of innovative products intended to (1) significantly reduce the risk of smoking-related disease compared with continued cigarette smoking and (2) be accepted by smokers as substitutes for cigarettes. The Tobacco Heating System (THS), developed by PMI and commercialized in more than 40 countries under the brand name IQOS, consists of tobacco sticks (eg, Marlboro *HeatSticks* in Japan), a holder, and a charger. The THS holder heats the tobacco stick for up to 6 minutes to a temperature not exceeding 350°C, which is too low to initiate combustion. The elimination of combustion allows the nicotine to be delivered to the THS user in a way that is similar to cigarettes while significantly reducing the production of and exposure to HPHCs [9].

Smoking cessation remains the best way to decrease the risk of developing smoking-related diseases; however, THS is meant as an alternative for those who would otherwise continue to smoke. The evidence available to date for THS has demonstrated that its aerosol contains significantly reduced levels of HPHCs, resulting in reduced exposure to HPHCs in volunteer healthy smokers who switched from cigarettes to THS, as assessed by measuring urinary biomarkers of exposure to selected HPHCs [38-41]. The magnitude of reduction was comparable to that observed in smokers who abstained from smoking, which has been referred to as the “gold standard” for the assessment of candidate reduced risk products [42,43]. Longer exposure studies of at least 6 months have shown favorable biological changes in smokers switching to THS, thereby reflecting improvement of several pathophysiological pathways that may eventually lead to the development of tobacco-related diseases, such as inflammation, oxidative stress, or endothelial dysfunction [44,45]. Preclinical data on gingival [46] and oral epithelial [47] human organotypic cultures showed minor histopathological alterations and minimal cytotoxicity upon THS aerosol exposure compared with exposure to cigarette smoke as well as a very significantly reduced overall impact, as illustrated by the measurement of inflammatory mediators and by transcriptomic and metabolomic analyses. Considering the available preclinical and clinical data on exposure reduction, it is conceivable to assume that the reduction in exposure to toxicants may lead to favorable changes in the outcome of a standard of care treatment of chronic generalized periodontitis for patients who switch to THS compared with those who continue smoking cigarettes.

## Methods

### Study

This is a randomized controlled two-arm parallel-group multicenter study comparing the treatment outcome of patients with chronic generalized periodontitis who switch from cigarettes to THS versus those who continue to smoke cigarettes for 6 months. This open-label study was conducted in 26 dental clinics in Japan and followed the principles defined in the International Conference on Harmonisation Guideline for Good Clinical Practice [48,49], Ministerial Ordinance on Good Clinical Practice for Drugs (Ministry of Health and Welfare, 1997 as last amended by the Ordinance of Ministry of Health, Labor and Welfare No. 9 of January 22, 2016) [50], Declaration of Helsinki [51], and other applicable regulations. Prior to the initiation of any study procedures, the protocol was approved at each site by the associated institutional review board. The study is registered in the US ClinicalTrials.gov registry with the identifier NCT03364751. The study was completed on December 15, 2018, and the data are currently being processed to obtain the results.

### Recruitment

This study enrolled current adult smokers of any brand of commercially available cigarettes who did not intend to quit smoking during the study and who had been diagnosed with chronic generalized periodontitis, as defined by the Japanese guidelines on periodontology [35]. Patients who were identified by dental practices participating in this study as potentially eligible were provided with information about the study, and if interested, they were invited to the screening visit (visit 1). At least 172 participants were planned to be randomized. Enrollment was stopped when this number was reached.

All patients included in the study were first advised that the best way of preventing further periodontal disease progression is to stop smoking as defined in the Japanese guidelines for periodontitis. From visit 1 onward, information on the risks of smoking and advice to quit smoking were given to all patients at every visit, including a debriefing of patients to address any intended or unintended beliefs they might have about IQOS to ensure that the patients had an accurate understanding of product risks, including an understanding that IQOS has not been demonstrated to be less harmful than cigarettes.

The main inclusion and exclusion criteria are listed in Textbox 1. In brief, participants needed to have at least a 5-year smoking history with an average of 10 cigarettes per day based on self-reporting and had to be Japanese. Study participants received information on the study prior to signing the informed consent form.

**Main inclusion and exclusion criteria.**

**Inclusion Criteria**

- Signed informed consent form
- Japanese ethnicity
- Aged≥30 years
- Smoked on average at least 10 commercially available cigarettes per day (no brand restriction) for at least 5 years prior to visit 1 based on self-reporting. Smoking status will be verified based on a urinary cotinine test (ie, cotinine≥200 ng/mL)
- Has at least 15 natural teeth, excluding the teeth that need to be extracted or whose mobility grade is ≥3
- Diagnosed with generalized chronic periodontitis (ie, more than 30% of diseased teeth with a pocket depth ≥4 mm), considering only teeth that do not need to be extracted or whose mobility grade is ≥3
- Does not intend to quit smoking during the study

**Exclusion criteria**

- Self-reported history of diagnosed systemic diseases (eg, stroke or acute cardiovascular event within the last 5 years, diabetes, active cancer) or any other conditions that in the opinion of the investigator would jeopardize the safety of the participant or affect the validity of the study results
- Has orthodontic appliances
- Received root planing therapy within the 6 months prior to visit 1
- Received surgical periodontal therapy within 3 years prior to visit 1
- Identifiable premalignant changes of the oral mucosa at visit 1
- Treated within the 3 months prior to visit 1 with systemic antibiotics or treated with topical antibiotics applied in the mouth
- Continuous systemic use of steroidal or nonsteroidal anti-inflammatory drugs for more than 20 days during the past 30-day period (except for low-dose aspirin, ≤300 mg, for prevention of thrombus/embolus in angina pectoris, myocardial infarction, transient ischemic cerebrovascular accidents, bypass operations, and similar)
- Women who are pregnant, breastfeeding, or planning a pregnancy within the course of the study

All subjects received financial compensation according to a payment scale agreed by the Institutional Review Board, which covered their time and transportation costs to and from the dental clinic. Additionally, patients who quit smoking or were using IQOS, or those who quit smoking and started using IQOS, would not forfeit their financial compensation.

### Study Design

#### Overview

**Figure 1:**
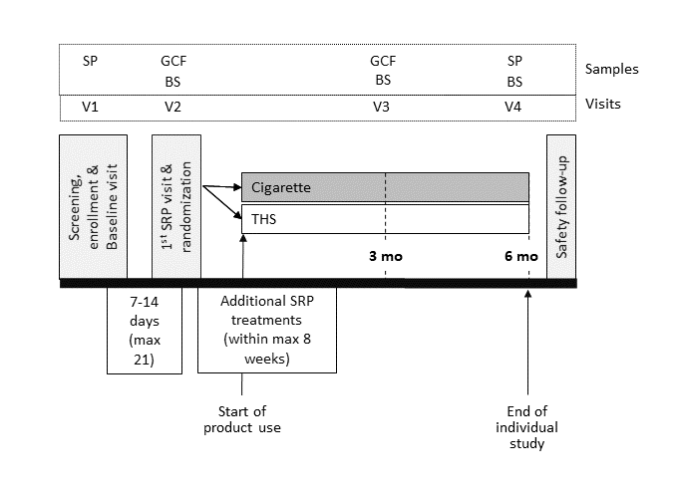
Design of the study. Eligibility criteria were checked at V1. Enrolled subjects were randomized at V2. Periodontal assessments were performed at V1, V3, and V4. Times of collection of buccal samples are indicated on the chart. BS: buccal swabs; GCF: gingival crevicular fluid; mo: month; SP: subgingival plaque; SRP: scaling and root planing; V: visit; THS: Tobacco Heating System.

The study design is illustrated in Figure 1, including four scheduled visits. From visit 2 to visit 4, the patients were asked not to drink, eat, chew gum, use mouthwash, or brush their teeth for at least 30 minutes before collection of oral samples (ie, subgingival plaque, gingival crevicular fluid, or buccal swabs).

#### Screening, Enrollment, and Baseline Visit (Visit 1)

Patients were invited to visit 1 by the investigator, following a standard visit to the investigator’s dentistry practice or by referral. During this visit, after signing the informed consent form, all eligibility criteria were checked, including periodontal assessments, which was also used as the baseline assessment for enrolled patients.

Concerning intention to quit, patients were questioned about their smoking history, and self-reported current tobacco and nicotine-containing tobacco product use over the past month at visit 1. Patients were also asked if they were planning to quit smoking during the study, after having been provided with smoking cessation advice. There was no specific questionnaire on motivation to quit, as no cessation arm was included in this study.

#### First SRP Treatment and Randomization Visit (Visit 2)

The first SRP treatment was performed on visit 2. Patients were treated using SRP as per the standard of care recommended by the Japanese Society of Periodontology [35]. The number of visits for these treatments was left up to the investigator to decide, as it can be quite variable between patients and between dentists (see section “Unscheduled Visits for Following Treatments”).

Patients were informed of their randomized study arm during visit 2 and could start using their allocated product immediately after the randomization.

This study was designed as an ad libitum study, without product use restriction, to mimic “real life” conditions as closely as possible. THS devices were distributed to the patients by the sites after randomization, and any variant of *HeatSticks* available on the Japanese market could be used. All patients were asked to buy their own cigarettes or *HeatSticks* according to their needs for the study. They were instructed to use their allocated product.

#### Unscheduled Visits for Following Treatments

The following SRP treatments were performed in subsequent visits after visit 2 as agreed between the site and the patient (“unscheduled visits” as per the protocol definition). The number and timing of these SRP visits were flexible, but all treatments had to be completed within 8 weeks after visit 2.

#### Investigational Period (Visit 3 and Visit 4)

The investigational period consisted of a visit at 3 and 6 months after the randomization visit (visit 3 and visit 4, respectively), during which all periodontal assessments were made.

#### Safety Follow-up Period

After the procedures of discharge, patients entered a 7-day safety follow-up period. Any nonserious adverse event that was ongoing during the safety follow-up period was to be followed up by the investigator during that period until it had been resolved, stabilized (ie, no worsening of the condition), or an acceptable explanation had been found (eg, a chronic condition). All serious adverse events were to be actively followed up by the investigator, despite their continuation after the end of the safety follow-up period, until their resolution, stabilization (ie, no worsening of the condition), or an acceptable explanation had been found (eg, a chronic condition).

### Randomization

The study followed a two-arm parallel randomization design with the following strata: (1) daily cigarette consumption over the month prior to visit 1 and visit 2; and (2) severity of disease (based on the size of PD) in smokers with generalized chronic periodontitis [35]. This selection was based on the fact that the degree of smoking exposure is related to the severity of periodontal destruction [5,52], and that the pretreatment PD and CAL have been shown to affect the response to therapy [53,54].

Randomization was performed through the Interactive Web/Voice Response System (IxRS) at visit 2. Patients were randomized into one of the two study arms (THS arm and cigarette arm) at a 1:1 ratio, using a stratified randomization based on daily cigarette consumption over the month (30 days) prior to visit 1 (10-19 cigarettes/day vs >19 cigarettes/day) and disease severity (<5 mm PD vs ≥5mm PD) in smokers with generalized chronic periodontitis. As per Japanese guidelines [35], disease severity is based on the tooth site having the most severe condition of PD [35].

### Blinding

Due to the nature of the exposure, blinding of the participants to the product was not possible. However, blinding of the examiners to the randomization arm of their patients was attempted, as described previously [16,24,55,56], to reduce the potential bias of the periodontal assessments that could be introduced by the smell of tobacco on patients using cigarettes, which is not present in patients using THS. If the examiner was unblinded, this had to be reported, but the patient was not discontinued from the study. Blinding was instead ensured by asking the patients to wash their hands before the examination, and the examiners all wore the same type of mask that neutralizes odors. Because mouthwash could influence the collection of samples, the patients were instructed not to use mouthwash during the study.

Additionally, even though limited, a certain degree of blinding has been applied during the study, including for data review. The clinical scientist and biostatistician will remain blinded to the subject randomization arm and actual CAL and PD values after randomization until database lock.

### Outcome Measures

All study objectives and endpoints are listed in Table 1. The choice of the primary and secondary objectives was based as a selection of (1) the most published periodontal endpoint (ie, reduction of PD, which has been shown to occur rather rapidly after mechanical therapy [within 3 to 6 months] and is more representative of the overall state of inflammation), (2) the most clinically relevant endpoint (ie, CAL change, which is more representative of tissue destruction and is thus a determinant of the increased risk of tooth loss), and (3) the most susceptible to change upon smoking cessation.

The assessment of both parameters, the primary objective for PD at 6 months and the secondary objective for CAL, will provide evidence on both the effect of using THS on inflammation status and on tissue repair, and will provide information about the modification of the healing profile related to switching to THS. All dental variables measured in this study were further selected based on the following criteria: (1) commonly assessed by dentists, (2) acceptability by patients, (3) robustness of the method (ie, index or evaluation criteria are available to assess improvement of periodontal disease), and (4) clinical relevance to support the objectives of the study. These variables include change in the gingival index score, change in tooth mobility (grade), change in plaque control record, and change in BOP scores.

**Table 1:** Objectives and endpoints of the study.

Objectives		Endpoints
**Primary objective**		
	To demonstrate the effect of switching to THS^a^ use compared to continued cigarette smoking on the response of PD^b^ to mechanical periodontal therapy	Mean PD reduction in all sites with initial PD≥4 mm after mechanical periodontal therapy (6 months)
**Secondary objectives**		
	To evaluate the effect of switching to THS use compared to continued cigarette smoking on the response of PD and CAL^c^ to mechanical periodontal therapy over time	Mean PD change in sites with initial PD≥4 mm after mechanical periodontal therapy (3 months only); mean CAL change in sites with initial PD≥4 mm after mechanical periodontal therapy (3 and 6 months)
	To evaluate the differences of periodontal parameters in the response to periodontal therapy in patients who switch to THS use compared with those who continue to smoke cigarettes	Change in mean full-mouth CAL (3 and 6 months); change in mean full-mouth PD (3 and 6 months); mean PD change in sites with initial PD<4 mm, 4-5 mm, 5-6 mm, 6-7 mm, and ≥7 mm (3 and 6 months); mean CAL change in sites with initial PD<4 mm, 4-5 mm, 5-6 mm, 6-7 mm, and ≥7 mm (3 and 6 months); change in the number of sites with PD<4 mm, 4-5 mm, 5-6 mm, 6-7 mm, and ≥7 mm (3 and 6 months); change in GI^d^ score (3 and 6 months); change in tooth mobility (grade) (3 and 6 months); change in PCR^e^ (3 and 6 months); change in BOP^f^ scores (3 and 6 months)
	To evaluate the levels of biomarkers of exposure over the exposure period in patients who switch to THS use and patients who continue to smoke cigarettes	Urinary nicotine equivalents, total 4-[methylnitrosamino]-1-[3-pyridyl]-1-butanol and 2-cyanoethylmercapturic acid (3 and 6 months)
	To describe self-reported tobacco or nicotine-containing product use over the duration of the study in patients switching to THS use and patients who continue to smoke cigarettes	Number of self-reported tobacco- or nicotine-containing product use
	To monitor safety	Incidence of adverse events/serious adverse events, including those related to device events (eg, device malfunction/misuse) over the duration of the study
**Exploratory objectives**		
	To determine quantitative changes in the inflammatory response by measuring inflammatory and immunoregulatory mediators in the GCF^g^ in patients switching to THS use compared with those continuing to smoke cigarettes	Measurement of proinflammatory and immunoregulatory mediators in the GCF (3 months)
	To evaluate the microbiological status in patients switching to THS use compared with those continuing to smoke cigarettes	Microbiological status from subgingival plaque samples (6 months)
	To evaluate the transcriptomics profile of buccal swabs in patients switching to THS use compared with those continuing to smoke cigarettes	Full transcriptomics profile assessment of buccal swabs derived from the right and left buccal mucosa (3 and 6 months)

^a^THS: Tobacco Heating System.

^b^PD: periodontal pocket.

^c^CAL: clinical attachment level.

^d^GI: gingival inflammation score.

^e^PCR: plaque control record.

^f^BOP: bleeding on probing.

^g^GCF: gingival crevicular fluid.

### Classification of Product Use Exposure

Although the patients were requested to use solely the product allocated to their respective study arm, it was likely that not all patients randomized to the THS or cigarette arms were going to be exclusive users of their randomized product at all times during the study. Patients were not excluded from the study if they concomitantly used THS and cigarettes or other tobacco- or nicotine-containing product (TNPs) available on the Japanese market (eg, heat-not-burn products other than THS, e-cigarettes, smokeless tobacco pipe, smokeless tobacco, cigars/pipes/kiseru/shisha, and NRT). However, as the participants in this study were cigarette smokers at enrollment, it was assumed that use of TNPs other than cigarettes or THS was going to be minimal over the investigation period. Thus, classification of patients according to a specific pattern of use will focus on cigarette and/or THS use only over the study duration.

**Figure 2:**
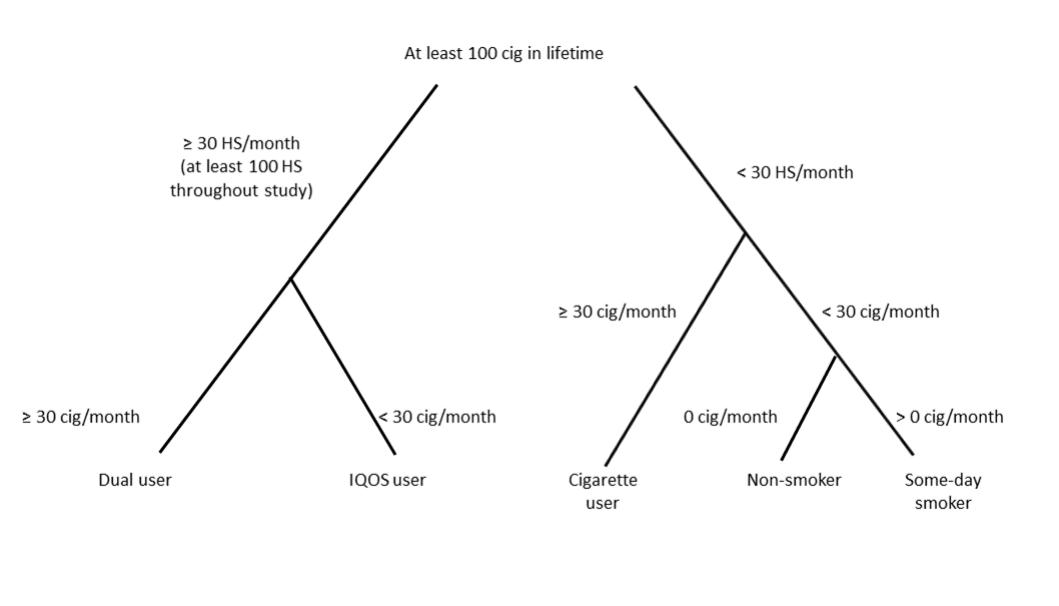
Product use classification tree. Cig: cigarette; HS: HeatSticks.

Product use pattern categories will be specified based on the average number of products used per month of each category (ie, *HeatSticks* or smoked cigarettes) as self-reported in the product use questionnaire over the study duration. Actual product use pattern categorization is described in Figure 2. As per the US Centers for Disease Control, an “every day” smoker is defined as an adult who has smoked at least 100 cigarettes in his or her lifetime and who now smokes every day. A “some-day” smoker is defined as an adult who has smoked at least 100 cigarettes in his or her lifetime, who now smokes but does not smoke every day. Based on the definition of a daily smoker, and when considering that one cigarette per day is still associated with an increased risk of developing a disease [57,58], a cigarette smoker in this study was defined as a patient using 30 cigarettes or more per month. Based on a comparable definition, a patient who is defined as a THS user would be an adult who has used at least 100 *HeatSticks* and who uses THS every day. Patients not included in the cigarette, THS, or dual user categories will be considered as a category “other,” and their data will not be analyzed.

### Study Hypothesis

The primary study hypothesis is that there will be a favorable difference in the mean of all sites with an initial PD≥4 mm at 6 months in exclusive THS users compared with smokers who continue to smoke cigarettes among patients with generalized chronic periodontitis.

### Statistical Analyses

#### Power

In the literature reviewed, the papers of Rosa et al [24] and Preshaw et al [23] describe the results of assessments comparable to the study described in this protocol, in that they include some requirements on the minimum PD for inclusion, comparison of smokers with former smokers, and means with SD or SEM and sample size.

For this study, because there are no available data on the effects of a heat-not-burn product on the improvement of periodontitis, it was therefore assumed that the cigarette arm will have similar results to those obtained in previous studies for current smokers, and that the results in the THS arm will be somewhat comparable to previous findings in former smokers.

Based on the published data and recommendations from clinical practice guidelines of the American Dental Association [59], the estimates with PD differences and SD between smokers and nonsmokers will be based on data assessed at 3, 9, and 12 months. With an α of 2.5%, power of 80%, SD of 0.5, and effect difference of 0.25 mm, the sample size needed in each arm was determined to be 64 patients. By considering a 25% dropout rate and product switching, 86 patients per arm (ie, 172 patients in total) were considered needed to adequately power the study.

#### Planned Analyses

The primary analysis will evaluate the change in PD from baseline, which will be calculated for each patient across all periodontal sites with a baseline PD>4 mm, resulting in one value per patient per endpoint for each visit (baseline, month 3, and month 6). The primary analysis will be performed on patients as per product use exposure (refer to the section on product categories) using a mixed model for repeated measurements. The model will include the PD change from baseline as the dependent variable, adjusting for daily cigarette consumption and disease severity at visit 1; full-mouth PD at visit 1; exposure group; and its interaction with visit. Clinical site will be included as a random effect. The interaction between cigarette consumption at visit 1 and product use, interaction between disease severity at visit 1 and product use, time since last SRP, number of target tooth/teeth extracted, number of nonmeasured target tooth/teeth extracted, and baseline tooth mobility will be assessed by a model selection algorithm using likelihood ratio tests.

The analysis of PD at 3 months and of CAL at 3 and 6 months will also be performed as secondary endpoints using a similar model with an adjustment for multiplicity for the secondary testing of CAL and PD.

Biomarkers of exposure will be analyzed as concentration data adjusted for creatinine on a logarithmic scale using mixed model repeated measures with the biomarkers of exposure geometric mean value as the dependent variable, adjusting for daily cigarette consumption at baseline, sex, visit, baseline biomarkers of exposure level, exposure group, and its interaction with visit. Clinical site will be included as a random effect. The modeling assumptions will be evaluated similarly to that in the primary analysis, except that time since last SRP, number of target tooth/teeth extracted, number of nonmeasured target tooth/teeth extracted, and baseline tooth mobility will not be included as covariates.

### Data and Sample Collection

#### Baseline Data Collection

Patient demographics (sex, date of birth, ethnicity) were collected at baseline. Periodontal assessments that were part of the eligibility criteria will be used as baseline measures for enrolled patients.

#### PD and CAL Measurements

The probe used in this study was a PCPUNC15 (#30) manufactured by Hu-Friedy. Measurements were recorded in 1-mm increments and were rounded to the nearest millimeter based on visual judgment. PD was measured as the distance from the gingival margin to which a probe penetrates the pocket.

Full-mouth PD was measured based on 6 sites per tooth (6-site measurement method: mesial, mid, and distal aspects of the buccal and palatal/lingual surfaces) at a pressure of approximately 20 *g* using the intended probe at visit 1, visit 3, and visit 4. At least 4 of the 6 sites per tooth had to be evaluated and have measurements recorded for the tooth to be included in the assessment.

When PD was not measurable on more than 2 sites, the assessment of the tooth was considered as missing, and all periodontal assessments for that tooth were excluded from the calculation of the means over all teeth. CAL was measured as the distance from an invariable reference point such as the cemento-enamel junction to the bottom of the pocket, also using the 6-site measurement method in the full mouth at visit 1, visit 3, and visit 4. As for PD, when CAL was not measurable on 1 or 2 of 6 sites on visit 1, the site(s) were skipped, but the tooth could still be included in the assessment. When CAL was not measurable on more than 2 sites, the whole tooth was skipped. The number of teeth lost during the study will be included as a covariate in the models of PD and CAL.

#### Other Dental Assessments

BOP in the full mouth was determined to assess inflammatory status in the pocket and was evaluated as a binary variable (YES or NO) at 6 sites per tooth at visit 1, visit 3, and visit 4. By gentle probing (approximately 20 *g* pressure), the site was assessed as YES if bleeding occurred within 30 seconds.

Tooth mobility was assessed according to the Miller classification [60], with grades from 0 to 3 (from less to more severe) corresponding to how much a tooth moves horizontally, in the full mouth at visit 1, visit 3, and visit 4.

The presence of plaque (based on the plaque control record) on individual tooth surfaces in the full mouth was assessed following the method of O’Leary et al [21], in which plaque retention in the dentogingival areas of the mesial, distal, facial, and lingual tooth surfaces is determined as a binary variable (YES or NO) recorded at visit 1, visit 3, and visit 4. A routine plaque disclosing agent was used at each clinical site.

For assessing the degree of gingival inflammation, 6 surfaces of each of the target teeth were rated according to the score developed by Löe and Silness [61], with grades from 0 to 3 (from less to more severe), based on visual evaluation of redness, edema, ulceration, and tendency for spontaneous bleeding.

#### Sample Collection

Subgingival plaque, buccal swab, and gingival crevicular fluid samples were collected from the participants. Collection of gingival crevicular fluid samples will allow for assessment of inflammatory markers in the oral environment, whereas the plaque samples were collected to evaluate changes in the periodontal microbiome over time after switching to THS compared with continuing to smoke cigarettes. Transcriptomics profiling of buccal swab samples will provide a complete picture of genes that are differentially regulated when switching to THS compared with cigarette smoking.

Collection of spot urine from participants should allow for evaluation of adherence to the allocated products by measuring biomarkers of exposure that can distinguish users of conventional cigarettes from users of smokeless tobacco products (such as THS). The biomarkers of exposure to be evaluated are 2-cyanoethylmercapturic acid (CEMA), total 4-(methylnitrosamino)-1-(3 pyridyl)-1-butanol (NNAL) or nicotine-derived nitrosamine ketone, and nicotine equivalents (NEQ). All values will be reported over the levels of urinary creatinine.

### Questionnaires

At visit 1, patients were asked to report how many cigarettes per day on average they had smoked over the last 5 years (smoking history) as well as for how many years they had smoked, including how many cigarettes per day they had smoked on average since they started smoking. Throughout the study, they were then asked to report monthly what TNPs they had used over the last month, and on average, how many per day.

## Results

The first subject was screened on November 8, 2017, and the last subject was out on December 14, 2018 (on December 21, 2018, when considering the safety follow-up period). Final results will be published in 2021.

## Discussion

### Expected Outcomes

Only a few longitudinal studies have assessed the outcome of periodontal disease after smoking cessation, but they indicate that the recovery in PD, and to a lesser extent in CAL, after nonsurgical treatment is more successful in those who quit smoking [23,24,56,62,63]. Studies in smokers, nonsmokers, and former smokers indicate that smoking cessation has a beneficial effect on the outcome of periodontal treatment, and thus that the negative impact of tobacco use on periodontal disease is reversible within a timeframe of a few weeks to a few years, depending on the clinical endpoint assessed [15,16,20,62,64-66]. In this study, the effect of switching from cigarette smoking to the use of THS will be evaluated, which will demonstrate whether or not it is beneficial on the improvement of periodontitis following a nonsurgical, mechanical treatment. The change of PD was chosen as the primary endpoint rather than the change of CAL, because data on CAL showing changes in smokers vs nonsmokers or quitters are sparser than data on PD and are not very consistent [13].

All other endpoints should provide data on the effect of switching to THS on oral health in general, including buccal inflammation and microbiome composition.

Biomarkers of exposure to CEMA, total NNAL, and NEQ will serve as indicators of overall exposure of the patients to cigarettes throughout the study, as they can provide a good estimate of the exposure to tobacco smoke, which should be significantly reduced in users of THS. Because nicotine is expected to be delivered with THS at levels comparable to that delivered via cigarettes, the levels of NEQ are not expected to be lower in THS users than cigarette users, but they will serve as an overall estimate of exposure to tobacco.

### Limitations

To demonstrate a favorable effect of switching to THS compared with continuing smoking cigarettes, patients should ideally be exclusive users. There are no data suggesting a minimum amount of cigarettes per day that would not trigger any detriment to the periodontal status. Product consumption is based on self-reporting; thus, there cannot be any strict verification that what the participants report is true. A chemical verification could in principle be performed by measuring urinary levels of biomarkers of exposure, but this would require collection of 24-hour urine to obtain a reliable result. Instead, spot urine was collected in this study and the biomarkers of exposure levels will be adjusted to creatinine to adjust urinary excretion rates. The reliability of spot urine, taken at different times during the day depending on when the patient went to the visit, will need to be evaluated, but may prove to be of reduced relevance compared to 24-hour urine collection.

The effect of nicotine on periodontitis has not been largely investigated; however, NRT is proposed to patients who are willing to quit smoking to reduce withdrawal effects [67-70]. There is no clear evidence that nicotine alone has a detrimental effect on the recovery of diseased gums [30]. Because THS delivers nicotine but greatly reduces the levels of other HPHCs, this study may help to differentiate the effects of nicotine from those of HPHCs on the improvement of periodontitis.

The measurement of PD or CAL can be quite variable, as described by Leroy et al [71]. This variability depends on (1) how the examiner makes the measurement, with the depth of penetration, angulation, and force applied being major factors of variability; and (2) the accuracy of the probe itself, which can vary even in the same batch from a production line. Intraexaminer reproducibility has nevertheless been shown to be high, with calibration and operator training, rather than operator experience, being fundamental for reproducibility [72].

Prior to the start of the study, the investigators attended a formal calibration training session to ensure and confirm that standardized measurements would be performed. The calibration session was designed to ensure a certain alignment of the different examiners in their periodontal assessment, as recommended for periodontal clinical research, as bias is easily introduced when several examiners take part in a study [73]. The weight applied by the probe was tested (20-25 *g*), as well as the measurement of PD, on 4 sites, and the ability to detect calculus. The number of different examiners in this study is unusually high when compared to other clinical studies on periodontitis; thus, whether intraexaminer variability will be an issue or not remains to be determined when analyzing the data. Only SRP was part of the care provided, unless otherwise decided by the investigator (eg, in the case of worsening of the condition). The same type of probe was used in each center. All dentists had to pass a Good Clinical Practice certification, and they were supported by Clinical Research Organization staff at every step of the protocol. The sample size was calculated based on former publications and was actually one of the largest samples ever tested in a longitudinal study on periodontitis and SRP.

### Comparison With Prior Work

Most studies performed to date on smoking and periodontitis have included cigarette smokers. Products such as heat-not-burn or e-cigarettes as alternatives to smoking have appeared relatively recently, and health-related outcome studies on these products are still extremely scarce. In 2016, a pilot study followed smokers with mild periodontitis who switched to e-cigarette use for up to 2 weeks, which found a statistically significant increase in gingival inflammation when tobacco smokers switched from smoking to vaping [31]. The authors did not report that smoking cessation leads to comparable effects [16,17,74], which is in alignment with the description that BOP and the inflammatory response associated with plaque accumulation are reduced or delayed in smokers compared to never smokers [14-16]. New studies on the effects of e-cigarettes and oral health are now being published; however, these are mainly cross-sectional studies and have not shown any effect of switching or of the effects on periodontal treatment [75-77]. A prospective study comparing the effect of full-mouth ultrasonic scaling on cigarette smokers, e-cigarette users, and nonsmokers with periodontitis found no significant difference in plaque control record, BOP, and PD at 3 and 6 months after treatment between the e-cigarette users and the nonsmokers, while the smokers had significantly higher scores of plaque control record and PD at the 6-month follow up [78]. Overall, these studies seem to indicate that e-cigarette users have a better periodontal status than cigarette users, but likely not as good as that of never smokers. This study is thus the first of its kind by enrolling an estimated number of smokers with chronic generalized periodontitis that should be sufficient to demonstrate whether THS can indeed favorably influence the improvement of diseased teeth after standard of care treatment compared with patients who continue to smoke cigarettes.

### Conclusions

This study is part of a multilayered assessment program designed to evaluate whether THS can potentially reduce the risk of smoking-related diseases relative to continued smoking. The results of this study will confirm whether the reduction in exposure to HPHCs, excluding nicotine, when switching from cigarettes to THS leads to statistically significant favorable changes in the improvement of periodontal pockets following mechanical therapy, and follows the direction expected upon smoking cessation. This study will provide further evidence to substantiate the reduced risk potential of THS.

## Abbreviations

BOP: bleeding on probing
CAL: clinical attachment level
CEMA: 2-cyanoethylmercapturic acid
HPHC: harmful and potentially harmful constituents
NEQ: nicotine equivalents
NNAL: 4-(methylnitrosamino)-1-(3 pyridyl)-1-butanol
NRT: nicotine replacement therapy
PD: pocket depth
PMI: Philip Morris International
SRP: scaling and root planing
THS: Tobacco Heating System
TNP: tobacco- or nicotine-containing product
